# Struvite Precipitation for Ammonia Nitrogen Removal in 7-Aminocephalosporanic Acid Wastewater

**DOI:** 10.3390/molecules17022126

**Published:** 2012-02-21

**Authors:** Zaixing Li, Xuguang Ren, Jiane Zuo, Yanfang Liu, Erhong Duan, Jingliang Yang, Ping Chen, Yongjun Wang

**Affiliations:** 1 School of Environmental Science and Engineering, Hebei University of Science and Technology, Shijiazhuang 050018, China; Email: Li_zaixing@163.com (Z.L.); 275716074@qq.com (X.R.); liuyanfang1984@163.com (Y.L.); 2 Department of Environmental Science and Engineering, Tsinghua University, Beijing 100084, China; Email: jiane.zuo@tsinghua.edu.cn; 3 North China Pharmaceutical Company, Shijiazhuang 050015, China; Email: chenping@ncpc.com (P.C.); wyjkx@163.com (Y.W.)

**Keywords:** struvite, 7-aminocephalosporanic acid, wastewater, ammonia nitrogen, precipitation

## Abstract

7-Aminocephalosporanic acid wastewater usually contains high concentrations of ammonium (NH_4_^+^-N), which is known to inhibit nitrification during biological treatment processes. Chemical precipitation is a useful technology to remove ammonium from wastewater. In this paper, the removal of ammonium from 7-aminocephalosporanic acid wastewater was studied. The optimum pH, molar ratio, and various chemical compositions of magnesium ammonium phosphate (MAP) precipitation were investigated. The results indicated that ammonium in 7-aminocephalosporanic acid wastewater could be removed at an optimum pH of 9. The Mg^2+^:NH_4_^+^-N:PO_4_^3^^−^-P molar ratio was readily controlled at a ratio of 1:1:1.1 to both effectively remove ammonium and avoid creating a higher concentration of PO_4_^3^^−^-P in the effluent. MgCl_2_·6H_2_O + 85% H_3_PO_4_ was the most efficient combination for NH_4_^+^-N removal. Furthermore, the lowest concentration of the residual PO_4_^3−^-P was obtained with the same combination. Struvite precipitation could be considered an effective technology for the NH_4_^+^-N removal from the 7-aminocephalosporanic acid wastewater.

## 1. Introduction

7-Aminocephalosporanic acid (7-ACA) is one of the key intermediates in the production of medically important semisynthetic cephalosporins, such as cephalaglycin and cephalothin. Currently, an enzyme-mediated process for the synthesis of 7-ACA from cephalosporin C has been recommended as an environmentally friendly technology compared to the conventional chemical synthetic process. During the enzyme-mediated process for the synthesis of 7-ACA, high levels of ammonium nitrogen (NH_4_^+^-N) and high chemical oxygen demand (COD) were found in the wastewater. NH_4_^+^-N present in wastewater at excess levels may deteriorate the receiving water quality [1]. In addition, NH_4_^+^-N is harmful to the local ecology [2]. Therefore, these compounds should be removed from the wastewater before entering into aquatic systems. However, the 7-ACA wastewater it is hard to bioremediate, because of the high concentrations of NH_4_^+^-N, a small quantity of cephalosporin and 7-ACA that can inhibit the growth of, and even destroy, harmful microorganisms. To overcome this difficulty, the precipitation of NH_4_^+^-N by forming magnesium ammonium phosphate (struvite, MgNH_4_PO_4_·6H_2_O) is an attractive means of 7-ACA wastewater treatment. NH_4_^+^-N recovered by sturvite may be reused as slow release fertilizer. Struvite crystallizes is a white orthorhombic crystalline structure consisting of magnesium, ammonium, and phosphate in equal molar concentrations [3]. The basic chemical reaction to form struvite is expressed in Equation (**1**) [4]:


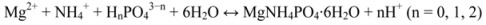
(1)

The method of chemical precipitation of NH_4_^+^-N in the form of struvite has been studied widely from various types of wastewaters such as landfill leachate [5], industrial wastewater [6], source-separated human urine [7], anaerobic swine lagoon liquid [8] and semiconductor wastewater [9]. Münch and Barr [10] have reported that the success of struvite precipitation depended on two main factors: Mg^2+^:NH_4_^+^-N:PO_4_^3−^-P ratio and the pH of the solution. Li and Zhao [11] found that under an equal molar ratio of Mg^2+^:NH_4_^+^-N:PO_4_^3−^-P, the NH_4_^+^-N concentration could quickly be reduced from 5,618 mg/L to 112 mg/L by pretreating the chemical precipitation. Uludag-Demirer [12] and co-workers treated dairy manure by struvite precipitation and demonstrated that over 92% of NH_4_^+^-N removal was possible by adding Mg^2+^ ions at a concentration higher than 0.06 M. Ryu [13] studied the struvite precipitation process in semiconductor wastewater at the field-scale and found that the optimum reaction for ammonium nitrogen removal occurred at a pH of 9.2. Marti [14] has reported that the struvite solubility decreases when the pH increases. 

Struvite precipitation has been considered an effective technology for NH_4_^+^-N removal. Previous studies have tested chemical precipitations and obtained several empirical parameters. The chemicals used as Mg^2+^ and PO_4_^3−^-P ions source along with the molar ratios of Mg^2+^:NH_4_^+^:PO_4_^3−^ adopted, the optimal pH values determined and the removal efficiencies achieved by struvite precipitation are summarized in Table 1. However, many reaction factors, such as pH, the molar ratio of Mg^2+^:NH_4_^+^-N:PO_4_^3−^-P, initial NH_4_^+^-N concentration and interfering ions that influence struvite precipitation, are less well studied, hampering the wide application of chemical precipitation. To the best of our knowledge, the feasibility of struvite precipitation in 7-ACA wastewater has not yet been studied. 

**Table 1:** Removal of NH_4_^+^-N and PO_4_^3^^−^-P by struvite precipitation from different wastewaters.

Type of the waste	Chemicals added	Amount of the chemicals Mg^2+^:NH_4_^+^-N: PO_4_^3−^-P	Initial concentrations (mg/L)		Removal (%)		pH	Ref.
			NH_4_^+^-N	COD	NH_4_^+^-N	COD		
Landfill leachates	MgCl_2_·6H_2_O + Na_2_HPO_4_·12H_2_O	1:1:1	2750	3720	92	NI	9	[5]
Industrial wastewater	Bittern + KH_2_PO_4_	1.6:0.6:1	110	NI	91	NI	9.6	[6]
Effluent of a sewage sludge anaerobic digester	MgCl_2_·6H_2_O + 85% H_3_PO_4_	1.5:1:1	749	936.4	89.35	39.78	9	[10]
Coking wastewater	MgCl_2_·6H_2_O + Na_2_HPO_4_·12H_2_O	1:1:1	500	200	88	NI	9.5	[15]
Effluent of UASB treating poultry manure wastewater	MgCl_2_·6H_2_O + KH_2_PO_4_	1:1:1	1318	1800	85.4	54	9	[16]
Effluent from the anaerobic treatment of the baker’s yeast industry	MgCl_2_·6H_2_O + Na_2_HPO_4_	1.1:1:1.1	735	NI	83	NI	9.2	[17]
Swine wastewater	MgCl_2_·6H_2_O + K_2_HPO_4_	1:1:1	844.5	2139	88	47	9	[18]

The objective of this study is to investigate the removal of NH_4_^+^-N by struvite precipitation from 7-ACA wastewater using different magnesium and phosphate sources. In the experiments, the evaluations were focused on the following objectives: (1) optimizing the effects of operating parameters, such as the pH, Mg^2+^:NH_4_^+^-N:PO_4_^3−^-P molar ratio and mixing time for the precipitate; (2) appraising the performance of struvite precipitation on residual PO_4_^3−^-P and COD removal; and (3) examining the physical properties of the struvite products.

## 2. Results and Discussion

### 2.1. Batch Testing with Nine Combinations of Chemicals

**Figure 1:**
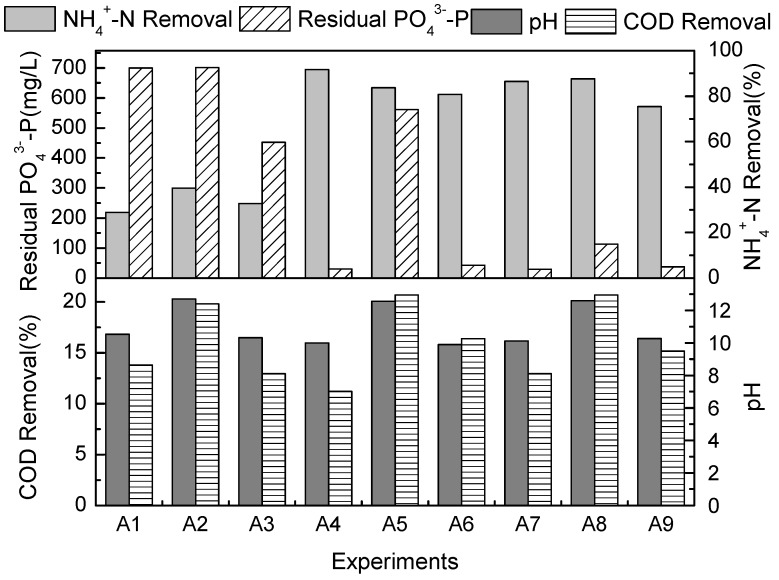
NH_4_^+^-N removal, residual PO_4_^3−^-P, pH and COD removal at a pH of 9, Mg^2+^:NH_4_^+^-N:PO_4_^3−^-P molar ratio of 1:1:1 and a mixing time 15 min.

In the first step of the struvite precipitation tests, nine combinations of chemicals denoted A1–A9 were tested with an initial NH_4_^+^-N concentration of 1,128 mg/L. Based on the stoichiometry of the struvite precipitation (Mg^2+^:NH_4_^+^-N:PO_4_^3−^-P = 1:1:1), the required quantities of chemicals were calculated and added to the 7-ACA wastewater. The overall performance of the precipitation reaction in terms of NH_4_^+^-N removal, COD removal, residual PO_4_^3−^-P in solution, and the change of pH is shown in Figure 1. When Mg^2+^ was added as MgO (experiments A1, A2 and A3) NH_4_^+^-N removal efficiencies were less than 40%. This phenomenon can be attributed to the fact that MgO has limited solubility in water. In addition, a high level of PO_4_^3−^-P was unexpectedly observed after the reaction, which is problematic because residual PO_4_^3−^-P will cause additional pollution in aquatic ecosystems. However, for MgCl_2_·6H_2_O and MgSO_4_ as alternate sources of Mg^2+^, NH_4_^+^-N removal efficiency increased up to 65%. Furthermore, the residual concentration of PO_4_^3−^-P was relatively low compared to that of the previous experiments. 

**Figure 2:**
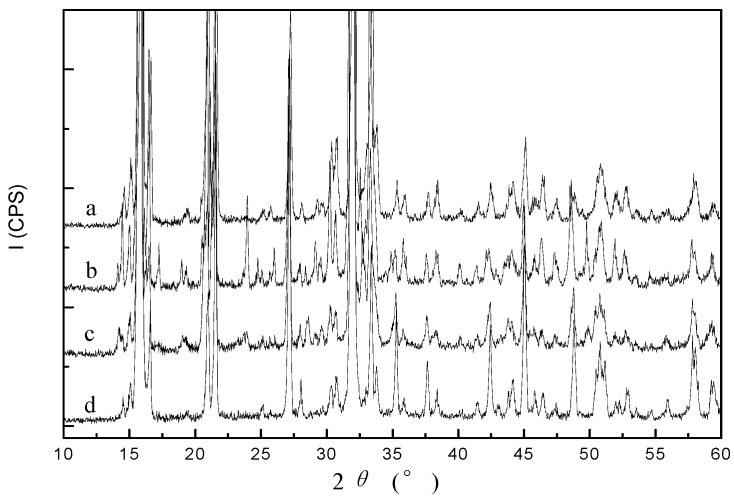
XRD diffractograms of precipitates for four chosen chemical combinations at pH 9 and a Mg^2+^:NH_4_^+^-N:PO_4_^3−^-P molar ratio of 1:1:1, (**a**) Na_3_PO_4_·12H_2_O + MgSO_4_; (**b**) NaH_2_PO_4_·12H_2_O + MgCl_2_·6H_2_O; (**c**) 85% H_3_PO_4_ + MgSO_4_; (**d**) 85% H_3_PO_4_ + MgCl_2_·6H_2_O.

**Figure 3:**
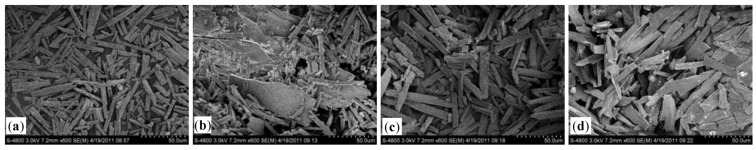
Morphology of struvite precipitations for four chosen chemical combinations at pH 9 and a Mg^2+^:NH_4_^+^-N:PO_4_^3^^−^-P molar ratio of 1:1:1 as analyzed via SEM: (**a**) Na_3_PO_4_·12H_2_O + MgSO_4_; (**b**) NaH_2_PO_4_·12H_2_O + MgCl_2_·6H_2_O; (**c**) 85% H_3_PO_4_ + MgSO_4_; (**d**) 85% H_3_PO_4_ + MgCl_2_·6H_2_O.

The addition of Na_3_PO_4_·12H_2_O + MgSO_4_ (A8), NaH_2_PO_4_·12H_2_O + MgCl_2_·6H_2_O (A5), 85%H_3_PO_4_ + MgCl_2_·6H_2_O (A4) or 85% H_3_PO_4_ + MgSO_4_ (A7) each achieved highly efficient removal of NH_4_^+^-N, with 70.92%, 67.83%, 74.28% and 70.02% of the total removed, respectively. To assess the quality of the struvite created through precipitation, the four combinations were analyzed by XRD and SEM analysis (Figure 2 and Figure 3). The XRD pattern generated from these samples matched the database model for struvite. The combination of 85% H_3_PO_4_ + MgCl_2_·6H_2_O showed the strongest match, indicating that a relatively pure precipitate of struvite could be created using 85% H_3_PO_4_ + MgCl_2_·6H_2_O. The results obtained from SEM morphological analysis were compared with the XRD analysis. As shown in Figure 3, the needle-shaped spherical crystal precipitate of 85% H_3_PO_4_ + MgCl_2_·6H_2_O was more distinct than the others, and its size was regular (radius 25–28 nm). Therefore, 85% H_3_PO_4_ + MgCl_2_·6H_2_O is proposed as the best condition to achieve maximum removal of NH_4_^+^-N from the 7-ACA wastewater.

The COD reduction was lower when compared with the corresponding NH_4_^+^-N removal fractions in the experiment (Figure 1), which implies that the struvite precipitation technique is highly selective for NH_4_^+^-N. This result is in good agreement with those reported by Li [11] and indicates that a subsequent biological treatment process may be needed to remove the residual COD.

The change in the pH of the solutions during the experiments was similar regardless of the choice of chemicals used. A decrease in pH value was observed in the course of the struvite reactions (Figure 1). Stratful [19] demonstrated that, in terms of thermodynamic equilibrium, hydrogen was released into the solution when struvite was formed, resulting in a decrease in pH. 

### 2.2. Effect of pH

**Figure 4:**
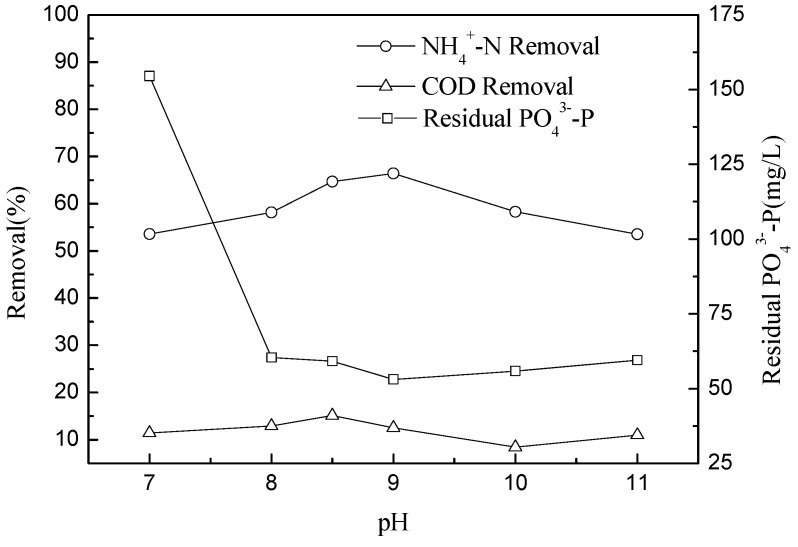
Effect of pH on NH_4_^+^-N and COD removals, residual PO_4_^3^^−^-P at Mg^2+^:NH_4_^+^-N:PO_4_^3^^−^-P molar ratio of 1:1:1 and mixing time 15 min.

pH is an important factor for struvite precipitation because it has a direct influence on the solubility of struvite and its thermodynamic properties [7]. The optimum pH for struvite precipitation has been widely investigated. In previous literature concerning struvite precipitation, optimum pH values of 8.5 [20,21], 9 [22], 8.9–9.25 [8], and 9.5–10.5 [23] were reported. In this study, to determine the optimum pH for NH_4_^+^-N removal in 7-ACA wastewater, the experiments were performed at a pH range of 7 to 11. Based on previous results, MgCl_2_·6H_2_O and 85% H_3_PO_4_ were used in subsequent batch experiments. The molar ratio of Mg^2+^:NH_4_^+^-N:PO_4_^3−^-P was at a stoichiometric ratio of 1:1:1. Figure 4 showed the obtained results.

**Figure 5:**
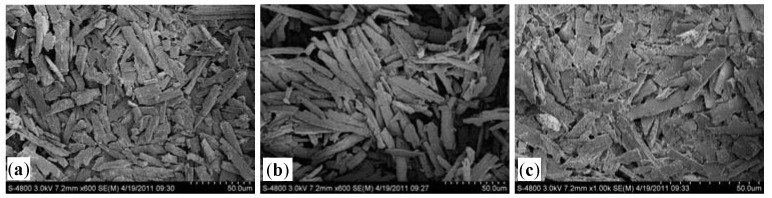
Morphology of struvite precipitation at different pH: (**a**) pH = 8 (**b**) pH = 9 (**c**) pH = 10.

Under otherwise constant precipitation conditions, changes in pH lead to a direct change in the degree of supersaturation during the precipitation process. At pH 7, no struvite was produced at detectable levels, while at pH 8, only a minute amount of very small crystals were produced. The growth of struvite crystals improved above pH 8, and the amount of precipitate at the bottom of beaker increased when the pH of the solution was gradually raised to 9. The struvite product was formed rapidly and settled quickly at the bottom of the beaker after stirring ceased at pH 9. However, the amount and the speed of formation of struvite precipitate decreased substantially at pH values of 10 and 11. Therefore, the best experimental ammonia removal was obtained at pH 9. At higher pH, the ammonia volatilization is serious. Air flow also plays an important role in ammonia-nitrogen volatilization. However, on the basis of the present experimental procedure (without stripping and only 15 min of stirring time) and also other findings in the literature [16,18], it can be concluded that ammonia volatilization is negligible on the removal of NH_4_^+^-N from the 7-ACA wastewater, as compared to struvite precipitation. It was likely that when the pH was excessively high, Mg_3_(PO_4_)_2_ was formed instead of struvite, which led to a decrease in the NH_4_^+^-N removal efficiency. H^+^ in the reaction solution should inhibit struvite precipitation when the pH is lower than the optimum point, which agrees with the reduced precipitation observed at lower pH. The optimum pH for the removal of ammonia observed in this experiment was consistent with other studies. Booker [24] reported that pH 9.2 was optimum, whereas Tünay [25] found pH 8.5–9.3 to be the optimal range. The morphology of struvite precipitation was observed both above and below the optimum pH of 9 (Figure 5).

Figure 4 also displayed the COD reduction and residual PO_4_^3−^-P for the 7-ACA wastewater. With an increase in pH, the percentage of COD removal maintained previous trends within a narrow range of 16–18%. The residual PO_4_^3−^-P in the 7-ACA wastewater was higher at pH < 8 than that at pH > 8 conditions. This may be because at low pH, further crystallization and precipitation of struvite was inhibited, and the residual concentration of PO_4_^3−^-P was maintained. The results indicate that the optimum pH values for the removal of ammonium and phosphate are different. This finding was consistent with the study of Booker [24] who reported that the maximum ammonium removal was found at pH 9.2, whereas the maximum phosphate removal was observed at pH 9.8.

Based on previous results, subsequent experiments were conducted at pH 9.0 with MgCl_2_·6H_2_O and 85% H_3_PO_4_ to investigate the effects of different molar ratios on the NH_4_^+^-N removal efficiency as well as on the residual PO_4_^3−^-P and COD.

### 2.3. Effect of the Mg^2+^:NH_4_^+^-N:PO_4_^3−^-P Molar Ratio

**Figure 6:**
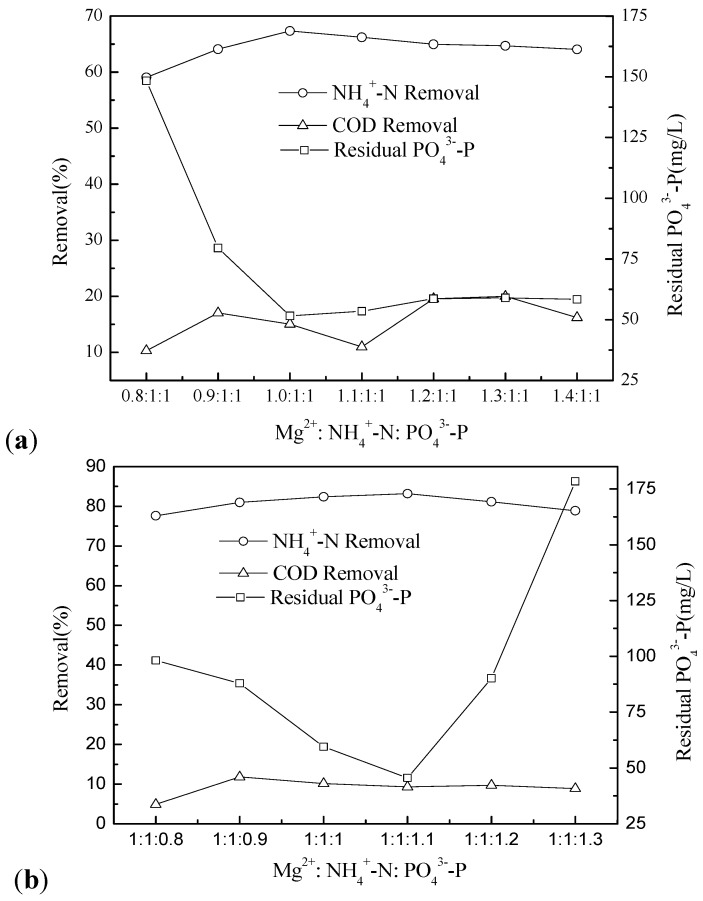
Effect of the mole ratio of Mg^2+^:NH_4_^+^-N:PO_4_^3−^-P on the removal of NH_4_^+^-N and the residual phosphate at pH 9.0 and mixing time 15 min, the molar ratio range of (**a**) Mg^2+^:NH_4_^+^-N:PO_4_^3−^-P = (0.8–1.3):1:1; (**b**) Mg^2+^:NH_4_^+^-N:PO_4_^3−^-P = 1:1:(0.8–1.3).

Effects of different Mg^2+^:NH_4_^+^-N:PO_4_^3−^-P molar ratios on NH_4_^+^-N removal, as well as on COD reduction and the residual PO_4_^3−^-P in the wastewater, were investigated for various molar concentrations. No significant improvement was observed in NH_4_^+^-N removal with increasing molar ratios of Mg^2+^:NH_4_^+^-N when the NH_4_^+^-N:PO_4_^3−^-P ratio was fixed at 1:1 (Figure 6a). This may be due to the formation of other precipitates at higher molar ratios. For example, when an excess concentration of Mg^2+^ is in highly alkaline conditions, solid phase Mg(OH)_2_ may precipitate. The precipitation of Mg_3_(PO_4_)_2_ may also occur because the precipitation potential of this compound is enhanced by the addition of additional Mg substrate. These results agree with the findings of several previous studies [25,26]. However, some scientists [3,27] have shown that NH_4_^+^-N removal was generally affected by the amount of magnesium available to the struvite precipitation reaction. In particular, Stratful [19] reported that magnesium ions were a limiting factor for struvite precipitation. The difference between these two contrary results may be due to the properties of the applied water.

The removal fraction of COD increased with an increasing concentration of Mg^2+^ species. COD removal reached 20.1% at the Mg^2+^:NH_4_^+^-N molar ratio of 1.3:1. Magnesium ions are widely used as flocculants to remove particulate organic matter in 7-ACA wastewater. The concentration of residual PO_4_^3−^-P first decreased and then increased with increasing Mg^2+^:NH_4_^+^-N ratio, indicating the existence of an optimum Mg^2+^: NH_4_^+^-N ratio for the removal of PO_4_^3−^-P.

The effect of the PO_4_^3−^-P: NH_4_^+^-N molar ratio was determined at a fixed Mg^2+^:NH_4_^+^-N ratio of 1:1 (Figure 6b). Theoretically, 100% of NH_4_^+^-N should be removed when the molar ratio of Mg^2+^:NH_4_^+^-N:PO_4_^3−^-P in the solution is equal to the stoichiometric value. However, the removal efficiency of NH_4_^+^-N was 81.3% when the PO_4_^3−^-P:NH_4_^+^-N ratio was 1:1. The removal efficiency of NH_4_^+^-N was increased a little with a rise of about 2.6% at the PO_4_^3−^-P:NH_4_^+^-N ratio of 1.1:1 and then decreased with the PO_4_^3−^-P:NH_4_^+^-N ratio above 1.1:1, but the removal efficiency remained lower than the theoretical value. Based on the wastewater characteristics and selected operating conditions, it may be possible to enhance the recovery of NH_4_^+^-N by adding excess concentrations of PO_4_^3−^-P. However, this application may be limited in practice due to excessively high levels of residual PO_4_^3−^-P after precipitation. As observed in Figure 6b, the concentration of residual PO_4_^3−^-P in the wastewater was substantially increased when the PO_4_^3−^-P:NH_4_^+^-N ratio was above 1.1:1. It is important to note that residual orthophosphate is itself a potential pollutant in the aquatic environment. 

The experimental results showed that NH_4_^+^-N removal reached nearly the maximum value at the stochiometric ratio. These results agreed with the findings of the previous study [27]. The residual PO_4_^3−^-P was higher when the Mg^2+^:NH_4_^+^-N:PO_4_^3−^-P at stochiometric ratio than the Mg^2+^:NH_4_^+^-N:PO_4_^3−^-P of 1:1:1.1. Taking into account the need to avoid excess residual PO_4_^3−^-P in the 7-ACA wastewater, the Mg^2+^:NH_4_^+^-N:PO_4_^3−^-P molar ratio of 1:1:1.1 was determined to be sufficient for the removal of NH_4_^+^-N from 7-ACA wastewater by struvite precipitation. 

### 2.4. Effect of Mixing Time

**Figure 7:**
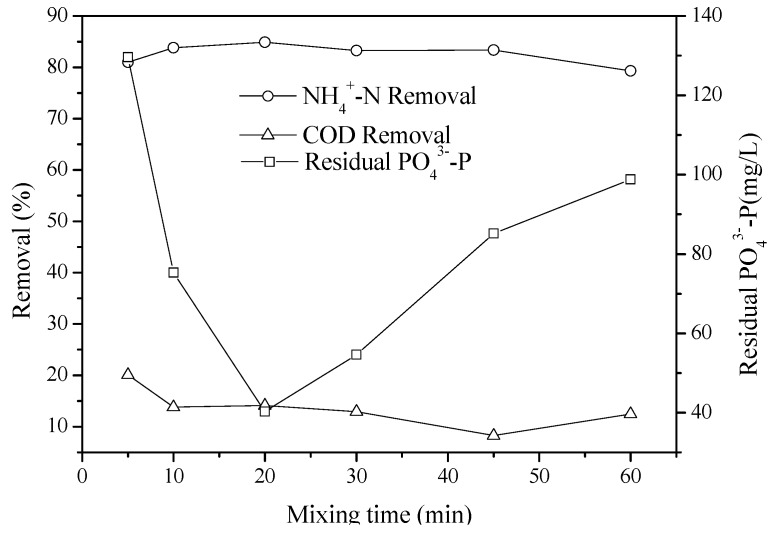
Effect of the mixing time on the removal of NH_4_^+^-N and the residual phosphate in the 7-ACA wastewater at pH 9 and the molar ratio of Mg^2+^:NH_4_^+^-N:PO_4_^3^^−^-P of 1:1:1.1.

**Figure 8:**
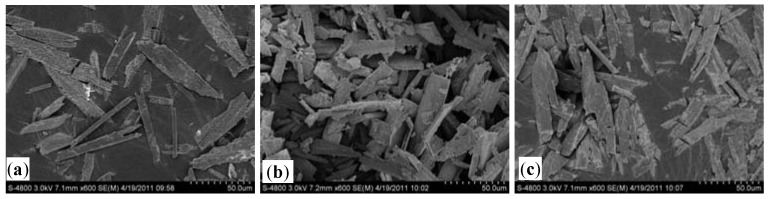
Morphology of struvite precipitation at various mixing times: (**a**) mixing time = 10 min; (**b**) mixing time = 20 min; (**c**) mixing time = 45 min.

Figure 7 describes the effect of mixing time on the removal of NH_4_^+^-N. The Mg^2+^:NH_4_^+^-N:PO_4_^3−^-P molar ratio was fixed at a ratio of 1:1:1.1, and the initial pH was 9.0. Overall removal of NH_4_^+^-N was observed to be similar at different mixing times. At short mixing times, the removal efficiency of NH_4_^+^-N was not significantly reduced. As the mixing time increased, the removal efficiencies of NH_4_^+^-N did not significantly increase. The mixing time between 5 and 60 min had a negligible effect on the production of struvite, suggesting that struvite crystals form homogeneously under these conditions and that precipitation is rapid. Examination of the precipitate by SEM microscopy revealed that the maximum crystal size increased with time (Figure 8). Crystals up to 20 μm were precipitated at 10 min. At a mixing time of 60 min, the maximum crystal size had increased, with some crystals reaching lengths of 75 μm. Stratful [19] also investigated the effect of reaction time on the precipitation of struvite and obtained the same conclusion. Some of the crystals were broken with the time increasing, because of the low strength of the crystal, which were shown in Figure 8. We also found that the precipitation system was impeded. The residual phosphate was lowest at mixing time 20 min (Figure 7). A little amount of phosphate may be released from the broking struvite when the mixing time more than 20 min. Kim [9] investigated the effect of mixing intensity and mixing duration on struvite precipitation and reported that mixing enhanced the transfer of mass from the solute to the crystals, resulting in improved struvite crystallization and growth.

## 3. Experimental

### 3.1. 7-ACA Wastewater

The 7-ACA wastewater used in this study was taken from an enzymatic transformation-based production line for antibiotics at a pharmaceutical plant in Hebei, China. The wastewater was generated from the oxidative deamination and hydrolysis catalyzed processes of 7-ACA manufacturing. The characteristics of the 7-ACA wastewater are summarized in Table 2. The analysis techniques used for the 7-ACA wastewater were in accordance with the Standard Method for the Examination of Water and Wastewater [28].

**Table 2:** Characteristics of 7-ACA wastewater.

Parameter	Concentration range
Total suspended solid (mg/L)	662 ± 97
COD (mg/L)	10850 ± 364
pH	12.2 ± 0.3
NH_4_^+^-N (mg/L)	1120 ± 82
PO_4_^3−^-P (mg/L)	36 ± 2
Turbidity (NTU)	71 ± 19
Biological oxygen demand (mg/L)	Under limitation

### 3.2. Reagents

For struvite formation, three different Mg^2+^ providing chemicals, namely MgO, MgCl_2_·6H_2_O and MgSO_4_, were compared in the experiments. H_3_PO_4_ (85%), Na_3_PO_4_·12H_2_O and NaH_2_PO_4_·2H_2_O were used as alternate sources of orthophosphate ions, and 8 M NaOH and 1 M NaOH were used to control pH in the solutions. All chemicals used were of analytical grade. 

### 3.3. Experimental Procedures

The experiments were performed at 298.15 K with a ZRS-6 variable-speed jar test apparatus (Tangshan Dachang Chemical Ltd., Tangshan, China). The jars were made of polytetrafluoroethene with dimensions of *Φ* 9.5 cm × 15 cm and held 1.0 L liquid. A two-blade propeller (polytetrafluoroethylene) with diameter of 2.5 cm and height of 7.6 cm was used for stirring.

Nine combinations of chemicals, including 85% H_3_PO_4_ + MgO (A1), Na_3_PO_4_·12H_2_O + MgO (A2), NaH_2_PO_4_·2H_2_O + MgO (A3), 85% H_3_PO_4_ + MgCl_2_·6H_2_O (A4), NaH_2_PO_4_·2H_2_O + MgCl_2_·6H_2_O (A5), Na_3_PO_4_·12H_2_O + MgCl_2_·6H_2_O (A6), 85% H_3_PO_4_ + MgSO_4_ (A7), Na_3_PO_4_·12H_2_O + MgSO_4_ (A8) and NaH_2_PO_4_·2H_2_O + MgSO_4_ (A9) were employed to select the best combination in terms of NH_4_^+^-N removal from 7-ACA wastewater.

Three factors that affect ammonium removal were studied: pH, the molar ratio of Mg^2+^:NH_4_^+^-N:PO_4_^3−^-P, and the mixing time. The detailed precipitation parameters are listed in Table 3. All batch experiments were performed in duplicate.

**Table 3:** Experimental conditions of struvite precipitation for the removal of NH_4_^+^-N (initial NH_4_^+^-N concentration of 1,128 mg/L).

Entry	pH	Molar ratio of Mg^2+^:NH_4_^+^-N:PO_4_^3−^-P	Amount of 85% H_3_PO_4_ + MgCl_2_·6H_2_O (g + g)	Mixing time (min)
1	7	1:1:1	12.5 + 6.1	15
2	8	1:1:1	12.5 + 6.1	15
3	8.5	1:1:1	12.5 + 6.1	15
4	9	1:1:1	12.5 + 6.1	15
5	10	1:1:1	12.5 + 6.1	15
6	11	1:1:1	12.5 + 6.1	15
7	9	0.8:1:1	10 + 6.1	15
8	9	0.9:1:1	11.3 + 6.1	15
9	9	1.1:1:1	13.8 + 6.1	15
10	9	1.2:1:1	15 + 6.1	15
11	9	1.3:1:1	16.3 + 6.1	15
12	9	1:1:0.8	12.5 + 4.9	15
13	9	1:1:0.9	12.5 + 5.5	15
14	9	1:1:1.1	12.5 + 6.7	15
15	9	1:1:1.2	12.5 + 7.3	15
16	9	1:1:1.3	12.5 + 7.9	15
17	9	1:1:1.1	12.5 + 6.7	5
18	9	1:1:1.1	12.5 + 6.7	10
19	9	1:1:1.1	12.5 + 6.7	20
20	9	1:1:1.1	12.5 + 6.7	30
21	9	1:1:1.1	12.5 + 6.7	60

The effectiveness of pH was investigated first. The test jar was filled with ammonia/phosphate solutions, and the pH was adjusted to the given values (from 7 to 11) in different jars using 1 mol·L^−1^ NaOH. The solutions were then stirred at 100 rpm for 15 min, followed by 30 min of quiescent settling. When the reaction time had elapsed, the pH was measured, and the precipitate that had formed was collected by double filtration through a 0.2 mm membrane filter. After filtration, concentrations of the NH_4_^+^-N, PO_4_^3−^-P and COD in solution were analyzed. 

The previous procedures were repeated for the other two factors. Based on the preliminary test results, subsequent experiments were then performed at the optimum pH (as found in the previous step) using the most efficient chemical combination. To maintain the stoichiometric molar ratio (1:1:1) needed for struvite precipitation, Mg^2+^ and PO_4_^3−^-P sources was added to ensure high removal efficiencies of NH_4_^+^-N. A Mg^2+^ source (MgCl_2_·6H_2_O) and a phosphate source (85% H_3_PO_4_) in solid phase were added to the beaker to adjust the molar ratio of Mg^2+^:NH_4_^+^-N:PO_4_^3−^-P. To test the effects of reaction time on the removal of NH_4_^+^-N, on COD and the residual PO_4_^3−^-P in wastewater, mixing times between 5–60 min were chosen.

### 3.4. Analytical Methods

COD, total suspended solids, NH_4_^3+^-N, PO_4_^3−^-P, turbidity and pH analyses were performed at the Water Quality Lab, as described in the Standard Method for the Examination of Water and Wastewater [29]. Crystal phases of the struvites were obtained by XRD (D/max 2500PC, Rigaku, Tokyo, Japan) with Cu Kα radiation of wavelength 0.154 nm in the range of 2*θ* = 10–80° with a scan speed of 1.2 °/min. The morphologies of the struvites were analyzed by SEM (S-4800I, Hitachi, Tokyo, Japan) at 3.0 keV, which was equipped with an energy dispersive analysis system of X-ray (EDS).

### 3.5. Observation and Identification of Crystals

The struvites were washed with distilled water through the membrane filter and dried at 303.17 K for 72 h. The crystal size was examined using an Olympus BH-2 light microscope with a camera attachment. X-ray diffraction using a Siemens D5000 diffractometer and monochrome CoKa radiation (40 kV, 30 mA) was used to determine the identity of the precipitate. Scans from 2 to 75° 2*θ* were recorded with a scan speed of 0.08° 2*θ* per min. The scan length was 0.02°, and the time constant was 15 s by reference to Card Socabin from Diffract AT.

## 4. Conclusions

Struvite precipitation was applied for the removal of NH_4_^+^-N from 7-ACA wastewater. Nine combinations of chemicals were used [85% H_3_PO_4_ + MgO (A1), Na_3_PO_4_·12H_2_O + MgO (A2), NaH_2_PO_4_·2H_2_O + MgO (A3), 85% H_3_PO_4_ + MgCl_2_·6H_2_O (A4), NaH_2_PO_4_·2H_2_O + MgCl_2_·6H_2_O (A5), Na_3_PO_4_·12H_2_O + MgCl_2_·6H_2_O (A6), 85% H_3_PO_4_ + MgSO_4_ (A7), Na_3_PO_4_·12H_2_O + MgSO_4_ (A8) and NaH_2_PO_4_·2H_2_O + MgSO_4_ (A9)] to determine the most efficient combination for NH_4_^+^-N removal. The effects of the operational parameters on struvite precipitation were also investigated. Based on the results of the experimental tests, the following conclusions could be drawn:

(1) MgCl_2_·6H_2_O + 85% H_3_PO_4_ was the most efficient combination for NH_4_^+^-N removal compared with the other chemical combinations studied. Furthermore, the lowest concentration of the residual PO_4_^3−^-P was obtained with the same combination.

(2) pH was an important parameter in the removal of NH_4_^+^-N from 7-ACA wastewater. The optimum pH for NH_4_^+^-N removal was clearly observed at pH 9, and a slightly higher pH would be required for efficient residual PO_4_^3−^-P removal.

(3) Excess quantities of Mg^2+^ and PO_4_^3−^-P were of little benefit to struvite formation. A Mg^2+^:NH_4_^+^-N:PO_4_^3−^-P molar ratio of 1:1:1.1 was sufficient for NH_4_^+^-N removal with the appropriate levels of residual PO_4_^3−^-P in the 7-ACA.
